# Disparate responses of tumour vessels to angiotensin II: tumour volume-dependent effects on perfusion and oxygenation

**DOI:** 10.1054/bjoc.2000.1229

**Published:** 2000-06-15

**Authors:** O Thews, D K Kelleher, P Vaupel

**Affiliations:** Institute of Physiology and Pathophysiology, University of Mainz, Duesbergweg 6, Mainz, D-55099, Germany

**Keywords:** angiotensin II, tumour vasculature, tumour blood flow, tumour perfusion, tumour oxygenation

## Abstract

Perfusion and oxygenation of experimental tumours were studied during angiotensin II (AT II) administration whereby the rate of the continuous AT II infusion was chosen to increase the mean arterial blood pressure (MABP) by 50–70 mmHg. In subcutaneous DS- sarcomas the red blood cell (RBC) flux was assessed using the laser Doppler technique and the mean tumour oxygen partial pressure (*p* O _2_) was measured polarographically using O _2_-sensitive catheter and needle electrodes. Changes in RBC flux with increasing MABP depended mainly on tumour size. In small tumours, RBC flux decreased with rising MABP whereas in larger tumours RBC flux increased parallel to the MABP. As a result of these volume-dependent effects on tumour blood flow, the impact of AT II on tumour *p* O _2_ was also mainly tumour volume-related. In small tumours oxygenation decreased with increasing MABP during AT II infusion, whereas in large tumours a positive relationship between blood pressure and O _2_ status was found. This disparate behaviour might be the result of the co-existence of two functionally distinct populations of tumour vessels. In small tumours, perfusion decreases presumably due to vasoconstriction of pre-existing host vessels feeding the tumour. In larger malignancies, newly formed tumour vessels predominate and seem not to have this vasoresponsive capability (lack of smooth muscle cells and/or AT receptors), resulting in an improvement of perfusion which is not tumour-related per se, but is due to the increased perfusion pressure. © 2000 Cancer Research Campaign


*British Journal of Cancer* (2000) **83**(2), 225–231
© 2000 Cancer Research Campaign
doi: 10.1054/ bjoc.2000.1229, available online at http://www.idealibrary.com on IDEAL®

### Disparate responses of tumour vessels to angiotensin II: tumour volume-dependent effects on perfusion and oxygenation

**O Thews, DK Kelleher and P Vaupel**

Institute of Physiology and Pathophysiology, University of Mainz, Duesbergweg 6, D-55099 Mainz, Germany

**Summary** Perfusion and oxygenation of experimental tumours were studied during angiotensin II (AT II) administration whereby the rate of the continuous AT II infusion was chosen to increase the mean arterial blood pressure (MABP) by 50–70 mmHg. In subcutaneous DS-sarcomas the red blood cell (RBC) flux was assessed using the laser Doppler technique and the mean tumour oxygen partial pressure ($pO_2$) was measured polarographically using $O_2$-sensitive catheter and needle electrodes. Changes in RBC flux with increasing MABP depended mainly on tumour size. In small tumours, RBC flux decreased with rising MABP whereas in larger tumours RBC flux increased parallel to the MABP. As a result of these volume-dependent effects on tumour blood flow, the impact of AT II on tumour $pO_2$ was also mainly tumour volume-related. In small tumours oxygenation decreased with increasing MABP during AT II infusion, whereas in large tumours a positive relationship between blood pressure and $O_2$ status was found. This disparate behaviour might be the result of the co-existence of two functionally distinct populations of tumour vessels. In small tumours, perfusion decreases presumably due to vasoconstriction of pre-existing host vessels feeding the tumour. In larger malignancies, newly formed tumour vessels predominate and seem not to have this vasoresponsive capability (lack of smooth muscle cells and/or AT receptors), resulting in an improvement of perfusion which is not tumour-related per se, but is due to the increased perfusion pressure. © 2000 Cancer Research Campaign

**Keywords**: angiotensin II; tumour vasculature; tumour blood flow; tumour perfusion; tumour oxygenation

Tumour hypoxia – a common phenomenon in clinical oncology – is an important factor known to be able to modulate the sensitivity of tumours to several non-surgical tumour treatment modalities, such as standard radiotherapy, chemotherapy with $O_2$-sensitive agents or photodynamic therapy (Bush et al, 1978; Freitas and Baronzio, 1991; Hall, 1994; Horsman, 1993). In addition, the oxygenation status seems to affect biological properties of tumours which appear to be relevant in terms of their malignant behaviour (e.g. metastatic or apoptotic potential) (Giaccia, 1996; Graeber et al, 1996). For this reason, tumour oxygenation is an independent parameter predicting for the long-term prognosis (local control, overall survival) of cancer patients (Brizel et al, 1996; Höckel et al, 1993; 1996; Nordsmark et al, 1996).

The oxygen deficiency found in tumours results presumably from an imbalance of oxygen supply to the tissue and the oxygen consumption of the cells (Vaupel, 1993; Gulledge and Dewhirst, 1996). One major reason for the insufficient $O_2$ transport to the tissue is the inadequate and chaotic microvascular network showing a series of morphological and functional abnormalities. In tumour microvessels, for instance, tortuosity, excessive branching, blind endings, lack of contractile elements (smooth muscle cells), as well as interrupted endothelial linings and basement membranes, can be found (Konerding et al, 1995). These vascular networks have no hierarchical organization (Konerding et al, 1995) and show significant arterio-venous shunt perfusion as well as temporal variations in red cell flux (short of vascular stasis) (Kimura et al, 1996) or even intermittent blood flow with temporary stasis (Chaplin and Hill, 1995). These functional abnormalities lead to an insufficient nutrient and oxygen supply of the tumour tissue (Braun et al, 1999; Dewhirst et al, 1998; Vaupel et al, 1989).

The inadequate blood flow through the tumour is relevant not only for the oxygenation status but also for the pharmacokinetics of anticancer agents. Studies analysing the spatial distribution of perfusion within a tumour showed pronounced heterogeneity which might compromise drug delivery to some regions of the tumour (Tozer et al, 1996).

For these reasons, many attempts have been undertaken with the aim of increasing tumour perfusion and oxygen supply to the cells to improve treatment efficacy of radio- and/or chemotherapy. Several possible mechanisms have been analysed for their capability of modulating tumour perfusion (Jain and Ward-Hartley, 1984; Vaupel et al, 1998; 2000): (i) vasoactive agents, (ii) rheologically active drugs, (iii) haemodilution, (iv) lowering interstitial hypertension, (v) mild (low-dose) hyperthermia, and (vi) agents reducing intermittent occlusion of tumour vessels (Jirtle, 1988; Kelleher et al, 1998; Powell et al, 1997).

When vasoactive drugs are considered, these can be used in two different ways to enhance tumour blood flow. Firstly, vasodilators might be suitable to bring about a dilation of tumour feeding vessels and, by reducing the vascular (geometric) resistance of the tumour, might lead to an increase in perfusion (as long as an adequate perfusion pressure can be maintained). Secondly, vasoconstrictive agents supplied systemically might, by causing an

*Received 12 October 1999*
*Revised 24 January 2000*
*Accepted 19 February 2000*

*Correspondence to*: O Thews increase in the perfusion pressure, lead to a redistribution of blood flow in favour of the tumour.

However, the efficacy of vasoactive drugs seems to be questionable due to the functional abnormalities of tumour microvessels. Vasodilators (e.g. NO-donating drugs) may only be effective on tumour vessels which are previously in a contracted state. However, many tumours lack vascular smooth muscle cells (Konerding et al, 1989*b*; 1989*c*). In addition, the low extracellular pH (Vaupel et al, 1989) and elevated levels of NO (Dworkin et al, 1995, Kennovin et al, 1994) found in tumours both lead to a maximal vasodilation. For these reasons the use of vasodilators (especially NO-donors) to improve tumour perfusion and/or oxygenation may not be appropriate (Shan et al, 1997). At the other extreme, increasing the perfusion pressure by a systemic vasoconstriction might only be effective if tumour vessels were not to react to the vasoconstrictive stimulus. It has been postulated that in many solid tumours the vascular system behaves like a chaotic network of rigid tubes. An increase in mean arterial blood pressure (MABP) should therefore result directly in an improvement of blood flow through the tissue and subsequently in a change of the $O_2$ and substrate supply to the tumour cells. Another aspect of using vasoconstrictive drugs for increasing blood pressure might be an improvement of the convective transport of macromolecules to the tumour cells. By increasing the blood pressure the extravasation of these molecules out of the vasculature into the interstitial space can be enhanced (e.g. Netti et al, 1999). An alternative measure to improve tumour perfusion is the application of rheologically active agents, which can reduce viscous resistance of the blood flowing through the tumour (Kelleher et al, 1998).

The aim of this study was to analyse the impact of the vasoconstrictive drug angiotensin II on perfusion and oxygenation of experimental tumours. In particular, the investigation assesses whether changes in perfusion pressure result in comparable changes in tumour blood flow (as would be expected for a system of rigid tubes), or whether vasoactive effects take place in the tumour vascular system.

### MATERIALS AND METHODS

### Animals and tumours

Studies were performed using DS-sarcomas implanted subcutaneously onto the hind foot dorsum of male Sprague-Dawley rats (body weight 180–250 g). Animals were allowed access to a standard diet (type 1324; Altromin, Lage, Germany) and water ad libitum prior to experiments. For tumour implantation 0.4 ml of DS-sarcoma ascites (approximately $10^4$ cells $\mu l^{-1}$) was injected. Within 6–12 days tumours grew as flat, spherical segments and replaced the subcutis and corium completely with volumes of 0.5–2.5 ml. Up to this volume the tumour grows without being limited by the restricted space of the subcutaneous tissue on the hind foot dorsum (as confirmed by the exponential growth observed) and without inducing pain or an elevated tissue pressure due to a non-compliant capsula. Volumes were determined by measuring the three orthogonal diameters of the tumour and using an ellipsoid approximation with the formula: $V = \pi/6 \times d_1 \times d_2 \times d_3$. From the volume-growth curves, the volume doubling time was calculated during exponential tumour growth. All experiments had previously been approved by the regional animal ethics committee and were conducted in accordance to the German Law for Animal Protection and the UKCCCR Guidelines (UKCCCR, 1998).

### Surgical procedures

When tumours reached the desired volume, rats were anaesthetized with sodium pentobarbital (40 mg $kg^{-1}$ i.p., Nembutal; Ceva, Paris, France). Throughout all experiments, animals lay supine on a heated operating pad with the rectal temperature maintained at 37.5–38.5°C. Animals breathed room air spontaneously. Mean arterial blood pressure (MABP) was continuously monitored through connection of an arterial catheter placed in the left carotic artery to a Statham pressure transducer (type P23 ID; Gould, Oxnard, CA, USA). A second catheter placed in the external jugular vein was used to continuously infuse angiotensin II.

### Angiotensin II infusion

Angiotensin II (AT II; Sigma, Deisenhofen, Germany) was dissolved in isotonic saline at a concentration of 6 $\mu g$ $ml^{-1}$. This solution was continuously infused over 30 min with the infusion rate being adjusted (0.5–2.0 $\mu g$ $min^{-1}$ $kg^{-1}$ body weight) to achieve an increase in MABP of 50–70 mmHg. Due to fast adaptation of the animals to AT II, the infusion rate had to be increased during the experiments in order to maintain the target increase in blood pressure.

### Oxygenation measurement

The spatial distribution of tumour oxygen tensions ($pO_2$) was measured polarographically using steel-shafted microelectrodes (outer diameter 300 $\mu m$) and $pO_2$ histography (Eppendorf, Hamburg, Germany; for more details of this method see Vaupel et al, 1991). A small midline incision was made in the skin covering the lower abdomen and the Ag/AgCl reference electrode placed between the skin and the underlying musculature. For tumour $pO_2$ measurement, a small incision was made into the skin overlying the tumour using a 24-gauge needle and the electrode advanced to a depth of approximately 1 mm. The electrode was then automatically moved through the tissue in pre-set steps with an effective step length of 0.7 mm (Vaupel et al, 1991). Approximately 100 $pO_2$ values were obtained from each tumour in up to 12 parallel electrode tracks with a horizontal distance of 1–3 mm. The oxygenation status of each tumour was described by the mean and median $pO_2$, as well as the fraction of $pO_2$ values $\leq$2.5 mmHg and $\leq$5 mmHg. Oxygenation studies of individual tumours were generally carried out in less than 20 min. Additionally, arterial blood gas analysis was performed immediately before and after tumour tissue $pO_2$ measurements, using a pH/blood gas analyser (Type 178; Ciba Corning, Fernwald, Germany) to ensure that animals had arterial blood gases within the physiological range during the measurement period.

For assessing temporal changes in tissue oxygenation, mean tumour $pO_2$ was measured continuously using polarographic catheter electrodes of the Clark type (Licox; GMS, Kiel, Germany). The $O_2$-sensitive cathode has a length of 5 mm and is placed in a $O_2$-permeable flexible catheter with an outer diameter of approximately 350 $\mu m$. For positioning of the electrode a catheter was inserted into the tumour along its long axis. After placing the electrode in the trocar, the catheter was withdrawn leaving the electrode in the tumour centre. Measured $pO_2$ values were averaged over a period of 1 min. Continuously measured $pO_2$ data were expressed relative to the $pO_2$ value immediately prior to the start of AT II infusion.

 © 2000 Cancer Research Campaign

### Laser Doppler flowmetry

Red blood cell (RBC) flux was measured using the laser Doppler technique (semiconductor laser diode, wavelength 780 nm, output power 1–2.5 mW, cut-off frequency 15 kHz, Oxford Array; Oxford Optronics, Oxford, UK). Details of this method have been described earlier by Kelleher et al (1995; 1998). In each tumour RBC fluxes were obtained from three different central and peripheral locations in the tissue using needle probes (Model MPM NP, o.d. 0.5 mm). For insertion of the probe a small incision was made with a 24-gauge needle in the skin covering the tumour, such that bleeding from the wound was kept to a minimum. Total backscatter was recorded continuously in order to optimize probe positioning and to ensure a constant probe location. In cases where flux artefacts due to alteration of probe position (e.g. as a result of movement) occurred (as indicated by a sudden change in total backscattered light), the flux values obtained from these channels were excluded from further analysis. At the end of each experiment, the probes were left in place and the animal was sacrificed by an overdose of anaesthetic to obtain the 'biological zero' laser Doppler signal (which was always < 15% of the RBC flux at $t = 0$ min). After subtracting the 'biological zero' value, data were expressed as relative RBC flux, and represent percentage values related to the flux measurement immediately prior to the AT II infusion. RBC flux measurements were performed 30 min before, during and for 30 min after AT II infusion.

As a measure of the resistance to flow, the relative tumour vascular resistance (TVR) was calculated from the ratio of the normalized mean arterial blood pressure and the RBC flux.

### Statistical analysis

Results are expressed as means ± standard error of the mean (SEM). Differences between various groups were assessed by the two-tailed Wilcoxon-test for unpaired samples. The significance level was set at $\alpha = 5\%$. For regression analysis, continuously measured signals were averaged for each 2 min interval and the correlation of two signals was calculated by the correlation coefficient ($r$).

### RESULTS

Angiotensin II (AT II) infusion led to a significant increase in the mean arterial blood pressure (MABP) of up to 200 mmHg. The effect was dose-dependent and disappeared within a few minutes when the infusion was stopped.

The change in MABP as a result of AT II infusion was accompanied by changes in RBC flux, the pattern of these changes being tumour volume-dependent. Figure 1 shows two examples of MABP and relative RBC flux in a small (volume = 0.57 ml, Figure 1A) and a large tumour (volume = 2.05 ml; Figure 1B) during intermittent infusion of AT II. In the small tumour, the AT II-induced increase in blood pressure was accompanied by a significant worsening of tumour perfusion as indicated by a decrease in RBC flux down to 50% of the pretreatment level (Figure 1A). This effect on tumour blood flow was completely reversible. In the case of the large tumour, increasing MABP led to a rise in tumour perfusion parallel to changes in blood pressure (Figure 1B).

In order to quantify the impact of various tumour volumes on the response of tumour perfusion to AT II, MABP and RBC flux values were averaged and correlated for every 2-min interval

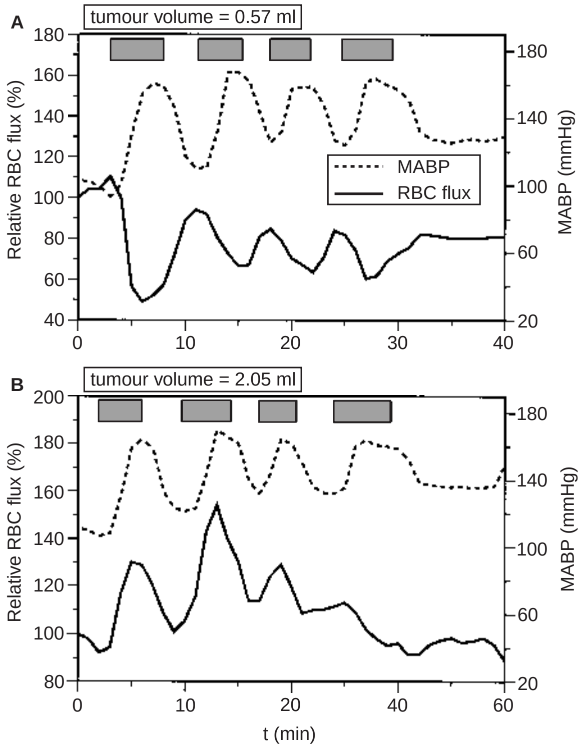

**Figure 1** Courses of MABP and RBC flux during intermittent application of angiotensin II in tumours of different sizes. (**A**) small tumour, volume = 0.57 ml. (**B**) large tumour, volume = 2.05 ml. Grey bars indicate periods of AT II infusion (demonstration of characteristic experiments).

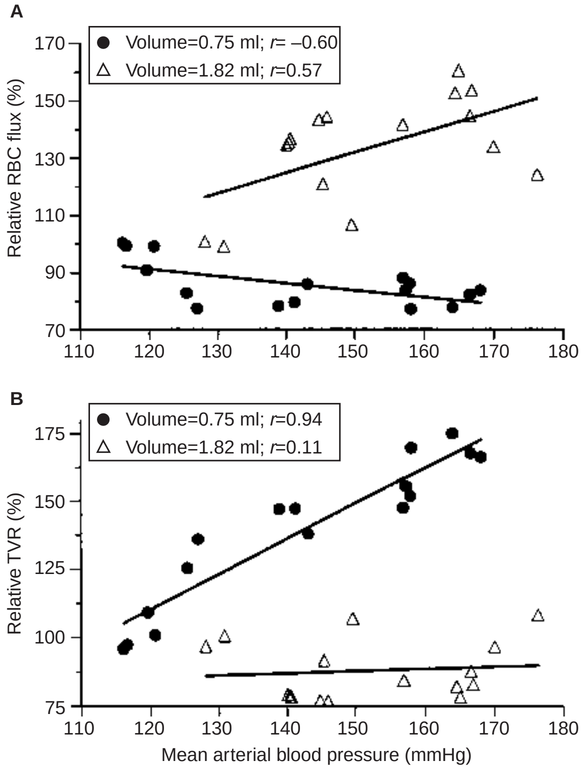

**Figure 2** Examples of the relationship between mean arterial blood pressure and (**A**) RBC flux, and (**B**) resistance to flow (TVR) during angiotensin II infusion in a small (volume = 0.75 ml) and a large (volume = 1.82 ml) tumour. Each point represents the mean value of a 2-min interval.

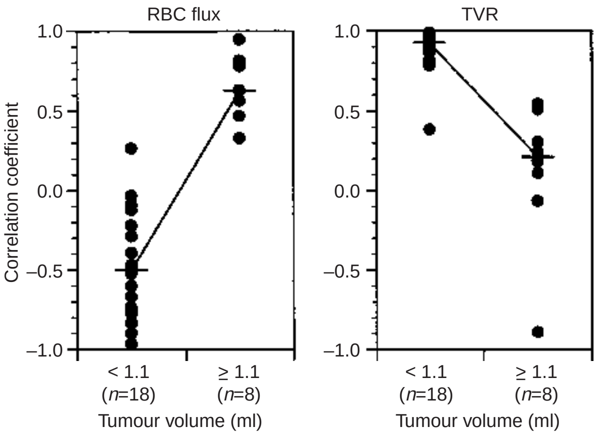

**Figure 3** Correlation coefficients of each tumour for the relationship between MABP and RBC flux and TVR, respectively, in different volume groups. The horizontal lines represent the median of the coefficients in each group (*n* = numbers of tumours investigated).

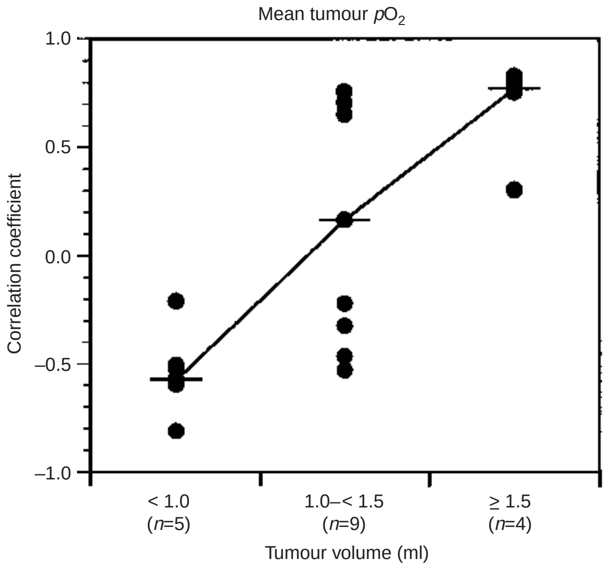

**Figure 5** Correlation coefficients of each tumour for the relationship between MABP and mean tumour $pO_2$ for different volume groups. The horizontal lines represent the median of the coefficients in each group (*n* = numbers of tumours investigated).

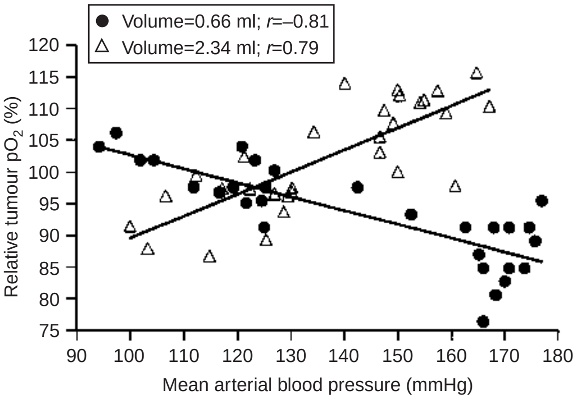

**Figure 4** Examples of the relationship between MABP and mean tumour $pO_2$ (normalized to pretreatment $pO_2$) before, during and after angiotensin II infusion in a small (volume = 0.66 ml) and a large (volume = 2.34 ml) tumour. Each point represents the mean value of a 2-min interval.

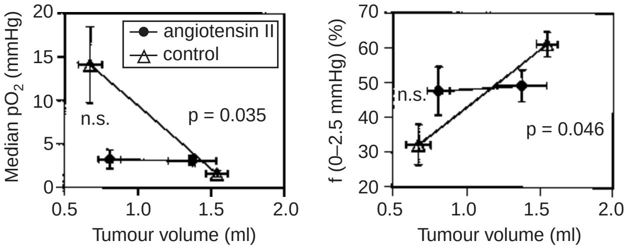

**Figure 6** Volume dependency of tumour oxygenation (expressed by the median $pO_2$ and the fraction of $pO_2$ measurements ≤2.5 mmHg) in control tumours (open triangles) and during continuous AT II infusion (dots). Each point represents a minimum of six tumours.

during AT II infusion. Figure 2 shows examples of the correlation between MABP and RBC flux and resistance to flow (TVR = MABP/RBC flux), for a small (volume = 0.75 ml) and a large (volume = 1.82 ml) tumour, respectively. Although the perfusion pressure increases during AT II application, the perfusion in the small tumour decreases ($r$ = –0.60) whereas the TVR significantly increases ($r$ = 0.94, Figure 2B). The large tumour shows a different behaviour. During AT II infusion, RBC flux increases with rising MABP ($r$ = 0.57, Figure 2A) and the resistance to flow (TVR) is almost unaffected by the AT II-induced increase in MABP ($r$ = 0.11, Figure 2B) indicating that the elevated tumour perfusion during AT II application is most probably the result of an increased perfusion pressure. On average, this behaviour of tumours of different sizes has been found in almost all tumours investigated. Figure 3 shows the correlation coefficients for each tumour separated for the different tumour volume groups.

Since perfusion is a paramount parameter determining the oxygenation status of tumours, the volume-dependent differences in tumour blood flow upon AT II application should be reflected in the $pO_2$ distribution in the tissue. In order to quantify the impact of AT II-induced changes on mean $pO_2$ in tumours of different volumes, $pO_2$ and MABP values were continuously monitored and averaged for every 2-min interval from 14 min before until 14 min after the end of AT II infusion. These averaged values of both parameters were then correlated. The initial $pO_2$ showed pronounced tumour volume-dependent differences (mean $pO_2$ = 31 ± 6 mmHg in small tumours with a volume < 1.0 ml, 30 ± 6 mmHg in medium-sized tumours with a volume of 1.0–< 1.5 ml and 9 ± 4 mmHg in large tumours with a volume ≥ 1.5 ml). For this reason, the measured $pO_2$ was normalized for each tumour to values prior to commencement of AT II infusion. Figure 4 shows two examples of the correlation between MABP and normalized tumour $pO_2$ for a small (volume = 0.66 ml) and a large (volume = 2.34 ml) tumour. In the small tumour, the mean $pO_2$ decreased with increasing MABP during angiotensin infusion ($r$ = –0.81) indicating an AT II-induced worsening of tumour oxygenation, presumably due to the observed decrease in RBC flux. In the larger tumour, the mean tumour $pO_2$ improved significantly parallel to the AT II-induced increase in MABP ($r$ = 0.79). Figure 5 shows the coefficients of all tumours investigated for the correlation between MABP and mean tumour $pO_2$. On average it could be demonstrated that in small tumours oxygenation worsens with

© 2000 Cancer Research Campaign increasing MABP (as indicated by a negative correlation coefficient) whereas in large tumours $pO_2$ increases with increasing MABP (positive correlation coefficient).

This tumour-volume-dependency of the angiotensin effect on tumour oxygenation is also reflected in the spatial $pO_2$ distribution. During AT II infusion the oxygenation of small tumours (volume < 1.1 ml) is worsened (expressed by a decrease in median $pO_2$ from 14 to 3 mmHg and an increase in the fraction of hypoxic $pO_2$ values ≤ 2.5 mmHg from 32 to 48%, Figure 6), whereas in larger tumours (volume ≥ 1.1 ml), the $O_2$ status was significantly improved. The spatial analysis of the $pO_2$ distribution showed that the improvement in large tumours is most pronounced in the tumour periphery, whereas more central regions remained almost completely hypoxic (as was the case in untreated control tumours).

### DISCUSSION

Newly formed tumour vessels show several structural and functional abnormalities (e.g. loss of vascular hierarchy, arterio-venous shunting) (Konerding et al, 1989a–c). In particular, the absence of a functioning innervation, smooth muscle cells and/or physiological receptors in these vessels results in an incapability to regulate perfusion to meet the tissue's demands (Mattsson et al, 1979; Peterson, 1983; 1991; Reinhold, 1979; Warren, 1979). At the same time however, normal vessels of the host tissue are incorporated into the growing tumour mass (Day, 1964; Mattsson et al, 1979; Warren, 1979). Thus, the rate and distribution of blood flow within a tumour reflects: (i) the fraction of newly formed tumour vessels as opposed to incorporated host vasculature; (ii) the arrangement of these different vessel populations within the tumour (parallel vs serial); and (iii) the structural organization of tumour vasculature relative to the surrounding (non-incorporated) normal tissue vessels, which may result in steal or anti-steal effects (Hirst, 1989; Vaupel, 1993). Vasoactive drugs affecting the functionally intact formerly host-tissue vessels can thus alter tumour perfusion in various ways. Where tumour vessels show no vasomotive responsiveness and are located parallel to the host-tissue vessels, a rise in MABP should increase tumour blood flow since in this case tumour vessels might react as a network of rigid tubes that will be better perfused due to the higher perfusion pressure.

For this reason, previous experiments have analysed the effects of (systemic) vasoconstriction on tumour perfusion and oxygenation. In early investigations adrenaline was used for inducing vasoconstriction. In these studies a reduction of tumour perfusion was usually observed (Cater et al, 1962; Gullino and Grantham, 1961; Mattsson et al, 1980). The use of AT II as a strong vasoconstrictor led to non-uniform results. In some studies AT II resulted in a passive expansion of the vascular bed due to the increased perfusion pressure (Hori et al, 1985; 1993), an increase in tumour perfusion (Shankar et al, 1999; Suzuki et al, 1981; 1984; Tokuda et al, 1990; Trotter et al, 1991), as well as a reduction in the fraction of transiently occluded microvessels (Hemingway et al, 1992; Trotter et al, 1991). In contrast, other studies showed a decrease in tumour perfusion during systemic application of AT II (Dworkin et al, 1997; Jirtle et al, 1978; Tozer and Shaffi, 1993; 1995; Tozer et al, 1996). Hirst et al (1991) demonstrated that the effect of AT II depended on the site of tumour implantation. In subcutaneously implanted tumours perfusion improved, whereas intra-abdominally growing tumours showed a decrease in blood flow. In the same way as perfusion, tumour oxygenation was also non-uniformly affected by AT II infusion (Suzuki et al, 1982).

In the present study, the impact of angiotensin on perfusion and oxygenation was tumour volume dependent. In small tumours (volume < 1.0 ml), both parameters worsened showing a negative correlation between the AT II-induced increase in MABP and RBC flux or tumour $pO_2$, respectively (Figures 2–5). In larger tumours (volume ≥ 1.5 ml), an improvement of perfusion and $O_2$ status with increasing MABP by AT II was seen (Figures 2–5). These tumour volume-dependent differences (in the same tumour model under identical experimental conditions) might be explained by the different responsiveness of tumour vessels upon AT II application in small and large tumours. Presumably, the AT II-induced increase in resistance to flow in small tumours (Figures 2B and 3) and the reduction in tumour perfusion is the result of a substantial vasoconstriction of the incorporated host vessels, i.e. the fraction of these functionally intact vessels is therefore responsible for the described net effect. In contrast, in large tumours, the perfusion seems to passively follow changes in systemic blood pressure according to Hagen–Poiseuille's law. This may reflect the overall inability of the vascular network to respond to AT II as seen by the lack of significant changes in TVR (Figures 2B and 3). Three reasons for the lack of reactivity of the tumour neovasculature to AT II have to be discussed: (i) many newly formed tumour vessels show no smooth muscle cells (Konerding et al, 1995; Warren, 1979), (ii) the density of AT II receptors is significantly reduced in these vessels compared with normal tissue vasculature (Andrade et al, 1998; Kohzuki et al, 1998; Tozer et al, 1996), and (iii) elevated levels of nitric oxide (NO) in these tumours (Kennovin et al, 1994). The third phenomenon might play a role since the DS-sarcoma shows a tumour-volume dependency of oxygenation leading to more pronounced hypoxia in larger tumours (Figure 6) which can in turn result in elevated levels of NO as compared to smaller tumours. NO has been shown to diminish pharmacologically induced vasoconstriction (Dworkin et al, 1995), which might explain the lack of responsiveness in larger tumours.

Differences in the density of AT II receptors might also be important for the spatial heterogeneity of the AT II-induced effect on tumour perfusion. Tozer et al (1996) described a significant decrease of tumour blood flow in peripheral areas of experimental P22 carcinosarcomas following AT II infusion, whereas perfusion in the tumour centre was almost unaffected. This pattern of spatial perfusion distribution upon AT II correlated well with the distribution of AT II receptor expression in tumour vessels found to be approximately 10% higher in the periphery (Tozer et al, 1996). The higher sensitivity of the tumour periphery to AT II might therefore be the reason for the observed vasoconstriction in these tumour areas. The authors also demonstrated that the change in tumour perfusion upon AT II, as well as the distribution of AT II receptors, show pronounced heterogeneity so that the overall (net) effect of AT II on tumour blood flow cannot be predicted.

As Hirst et al (1991) demonstrated, the site of tumour growth plays an important role in the responsiveness of tumour vessels to AT II. The DS-sarcoma used in the present study also shows differences in the vascular pattern when implanted into different host tissues (Vaupel and Gabbert, 1986) which in turn influences the nutrient supply and tumour oxygenation (Vaupel and Gabbert, 1986; Vaupel and Mueller-Klieser, 1986). However, if DS-sarcomas were implanted into the same host tissue (subcutis) but at different locations (e.g. hind foot dorsum, thigh), these parameters showed comparable values. For this reason, the results from the site of tumour growth used in the present study should be comparable to other s.c. locations used in experimental studies. Although

© 2000 Cancer Research Campaign differences in the vascular pattern of the tumour depending on the host tissue have been demonstrated, the present study clearly shows that under well-defined experimental conditions (same tumour model, same site of implantation) the responsiveness of the tumour vasculature to AT II strongly depends on the tumour volume.

Since in larger tumours perfusion increases with rising blood pressure, it can be concluded that in DS-sarcomas newly formed tumour vessels and pre-existing host-tissue vessels are arranged mostly parallel to each other. In small tumours however, functionally intact vessels appear to be located proximately to the tumour vessel network, because in these tumours AT II-induced vasoconstriction led to a worsening in tumour perfusion. Obviously, the fraction of functionally insufficient vessels from neovascularization increases during tumour growth whereas the (relative) number of integrated normal host-tissue vessels is reduced. From the results of the present study, it can be concluded that improving perfusion and oxygenation by systemic vasoconstriction is only possible in some tumours. Since the behaviour of tumour vessels to vasoconstrictive agents cannot be predicted a priori (Tozer et al, 1996), the applicability of these drugs in the clinical setting is questionable.

### ACKNOWLEDGEMENTS

This study was supported by a grant from the Deutsche Krebshilfe (70-1920-Va 2) and the Dr med. h.c. Erwin Braun Foundation, Basel, Switzerland.

### REFERENCES

Andrade SP and Beraldo WT (1998) Pharmacological reactivity of neoplastic and non-neoplastic associated neovasculature to vasoconstrictors. *Int J Exp Pathol* **79**: 425–432

Braun RD, Lanzen JL and Dewhirst MW (1999) Fourier analysis of fluctuations of oxygen tension and blood flow in R3230Ac tumors and muscle in rats. *Am J Physiol* **277**: H551–H568

Brizel DM, Scully SP, Harrelson JM, Layfield LJ, Bean JM, Prosnitz LR and Dewhirst MW (1996) Tumor oxygenation predicts for the likelihood of distant metastases in human soft tissue sarcoma. *Cancer Res* **56**: 941–943

Bush RS, Jenkin RDT, Allt WEC, Beale FA, Dembo AJ and Pringle JF (1978) Definitive evidence for hypoxic cells influencing cure in cancer therapy. *Br J Cancer* **37** (Suppl. 3): 302–306

Cater DB, Grigson CMB and Watkinson DA (1962) Changes of oxygen tension in tumours induced by vasoconstrictor and vasodilator drugs. *Acta Radiol* **58**: 401–436

Chaplin DJ and Hill SA (1995) Temporal heterogeneity in microregional erythrocyte flux in experimental solid tumours. *Br J Cancer* **71**: 1210–1213

Day ED (1964) Vascular relationships of tumor and host. *Prog Exp Tumor Res* **4**: 57–97

Dewhirst MW, Braun RD and Lanzen JL (1998) Temporal changes in $PO_2$ of R3230AC tumors in Fischer-344 rats. *Int J Radiat Oncol Biol Phys* **42**: 723–726

Dworkin MJ, Carnochan P and Allen-Mersh TG (1995) Nitric oxide inhibition sustains vasopressin-induced vasoconstriction. *Br J Cancer* **71**: 942–944

Dworkin MJ, Carnochan P and Allen-Mersh TG (1997) Effect of continuous regional vasoactive agent infusion on liver metastasis blood flow. *Br J Cancer* **76**: 1205–1210

Freitas I and Baronzio GF (1991) Tumor hypoxia, reoxygenation and oxygenation strategies: Possible role in photodynamic therapy. *J Photochem Photobiol* **11**: 3–30

Giaccia AJ (1996) Hypoxic stress proteins: survival of the fittest. *Seminars in Radiation Oncology* **6**: 46–58

Graeber TG, Osmanian C, Jacks T, Housman DE, Koch CJ, Lowe SW and Giaccia AJ (1996) Hypoxia-mediated selection of cells with diminished apoptotic potential in solid tumours. *Nature* **379**: 88–91

Gulledge CJ and Dewhirst MW (1996) Tumor oxygenation: A matter of supply and demand. *Anticancer Res* **16**: 741–750

Gullino PM and Grantham FH (1961) Studies on the exchange of fluids between host and tumor. II. The blood flow of hepatomas and other tumors in rats and mice. *J Natl Cancer Inst* **27**: 1465–1491

Hall EJ (1994) *Radiobiology for the Radiologist*. Lippincott: Philadelphia

Hemingway DM, Angerson WJ, Anderson JH, Goldberg JA, McArdle CS and Cooke TG (1992) Monitoring blood flow to colorectal liver metastases using laser Doppler flowmetry: the effect of angiotensin II. *Br J Cancer* **66**: 958–960

Hirst DG (1989) Tumor blood flow modification therapeutic benefit: is this approach ready for clinical application? Review. In *Gray Laboratory 1989 Annual Report*, Michael B, Hance M (eds) pp 14–17. Cancer Research Campaign: London

Hirst DG, Hirst VK, Shaffi KM, Prise VE and Joiner B (1991) The influence of vasoactive agents on the perfusion of tumours growing in three sites in the mouse. *Int J Radiat Biol* **60**: 211–218

Höckel M, Knoop C, Schlenger K, Vomdran B, Baussmann E, Mitze M, Knapstein PG and Vaupel P (1993) Intratumoral $pO_2$ predicts survival in advanced cancer of the uterine cervix. *Radiother Oncol* **26**: 45–50

Höckel M, Schlenger K, Aral B, Mitze M, Schäffer U and Vaupel P (1996) Association between tumor hypoxia and malignant progression in advanced cancer of the uterine cervix. *Cancer Res* **56**: 4509–4515

Hori K, Suzuki M, Abe I, Saito S and Sato H (1985) Increase in tumor vascular area due to increased blood flow by angiotensin II in rats. *J Natl Cancer Inst* **74**: 453–459

Hori K, Zhang QH, Saito S, Tanda S, Li HC and Suzuki M (1993) Microvascular mechanisms of change in tumor blood flow due to angiotensin II, epinephrine, and methoxamine: a functional morphometric study. *Cancer Res* **53**: 5528–5534

Horsman MR (1993) Hypoxia in tumours: its relevance, identification, and modification. In *Medical Radiology. Current topics in clinical radiobiology* Beck-Bomholdt HP (ed) pp. 99–112. Springer: Berlin

Jain RK and Ward-Hartley (1984) Tumor blood flow – characterization, modifications, and role of hyperthermia. *IEEE Trans Son Ultrason* **SU-31**: 504–526

Jirtle RL (1988) Chemical modification of tumour blood flow. *Int J Hyperthermia* **4**: 355–371

Jirtle R, Clifton KH and Rankin JH (1978) Effects of several vasoactive drugs on the vascular resistance of MT-W9B tumors in W/Fu rats. *Cancer Res* **38**: 2385–2390

Kelleher DK, Engel T and Vaupel PW (1995) Changes in microregional perfusion, oxygenation, ATP and lactate distribution in subcutaneous rat tumours upon water-filtered IR-A hyperthermia. *Int J Hyperthermia* **11**: 241–255

Kelleher DK, Thews O and Vaupel P (1998) Regional perfusion and oxygenation of tumors upon methylxanthine derivative administration. *Int J Radiat Oncol Biol Phys* **42**: 861–864

Kennovin GD, Flitney FW and Hirst DG (1994) ‘Upstream’ modification of vasoconstrictor responses in rat epigastric artery supplying an implanted tumour. *Adv Exp Med Biol* **345**: 411–416

Kimura H, Braun RD, Ong ET, Hsu R, Secomb TW, Papahadjopoulos D, Hong K and Dewhirst-MW (1996) Fluctuations in red cell flux in tumor microvessels can lead to transient hypoxia and reoxygenation in tumor parenchyma. *Cancer Res* **56**: 5522–5528

Kohzuki M, Tanda S, Hori K, Yoshida K, Kamimoto M, Wu XM and Sato T (1998) Endothelin receptors and angiotensin II receptors in tumor tissue. *J Cardiovasc Pharmacol* **31** (Suppl. 1): S531–S533

Konerding MA, Steinberg F and Budach V (1989*a*) The vascular system of xenotransplanted tumors – scanning electron and light microscopic studies. *Scanning Microsc* **3**: 327–335

Konerding MA, Steinberg F and Streffer C (1989*b*) The vasculature of xenotransplanted human melanomas and sarcomas on nude mice. I. Vascular corrosion casting studies. *Acta Anat (Basel)* **136**: 21–26

Konerding MA, Steinberg F and Streffer C (1989*c*) The vasculature of xenotransplanted human melanomas and sarcomas on nude mice. II. Scanning and transmission electron microscopic studies. *Acta Anat (Basel)* **136**: 27–33

Konerding MA, Miodonski AJ and Lametschwandtner A (1995) Microvascular corrosion casting in the study of tumor vascularity: a review. *Scanning Microsc* **9**: 1233–1243

Mattsson J, Alpsten M, Appelgren L and Peterson HI (1980) Influence of noradrenaline on local tumor blood flow. *Eur J Cancer* **16**: 99–102

Mattsson J, Appelgren L, Hamberger B and Peterson HI (1979) Tumor vessel innervation and influence of vasoactive drugs on tumor blood flow. In *Tumor Blood Circulation* Peterson HI (ed) pp 129–135. CRC Press: Boca Raton

 © 2000 Cancer Research Campaign

Netti PA, Hamberg LM, Babich JW, Kierstead D, Graham W, Hunter GJ, Wolf GL, Fischman A, Boucher Y and Jain RK (1999) Enhancement of fluid filtration across tumor vessels: implication for delivery of macromolecules. *Proc Natl Acad Sci USA* **96**: 3137–3142

Nordsmark M, Overgaard M and Overgaard J (1996) Pretreatment oxygenation predicts radiation response in advanced squamous cell carcinoma of the head and neck. *Radiother Oncol* **41**: 31–39

Peterson HI (1983) Innervation von Tumorgefässen, Einfluss vasoaktiver Substanzen und Regulation der Tumordurchblutung. *Mikrozirk Forsch Klin* **2**: 69–77

Peterson HI (1991) Modification of tumour blood flow – a review. *Int J Radiat Biol* **60**: 201–210

Powell ME, Hill SA, Saunders MI, Hoskin PJ and Chaplin DJ (1997) Human tumor blood flow is enhanced by nicotinamide and carbogen breathing. *Cancer Res* **57**: 5261–5264

Reinhold HS (1979) In vivo observations of tumor blood flow. In *Tumor Blood Circulation* Peterson HI (ed) pp 115–128. CRC Press: Boca Raton

Shan SQ, Rosner GL, Braun RD, Hahn J, Pearce C and Dewhirst MW (1997) Effects of diethylamine/nitric oxide on blood perfusion and oxygenation in the R3230Ac mammary carcinoma. *Br J Cancer* **76**: 429–437

Shankar A, Loizidou M, Bumstock G and Taylor I (1999) Noradrenaline improves the tumour to normal blood flow ratio and drug delivery in a model of liver metastases. *Br J Surg* **86**: 453–457

Suzuki M, Hori K, Abe I, Saito S and Sato H (1981) A new approach to cancer chemotherapy: selective enhancement of tumor blood flow with angiotensin II. *J Natl Cancer Inst* **67**: 663–669

Suzuki M, Hori K, Abe I, Saito S and Sato H (1982) Selective increase in tumor oxygen tension with angiotensin II. *Invasion Metastasis* **2**: 33–39

Suzuki M, Hori K, Abe I, Saito S and Sato H (1984) Functional characterization of the microcirculation in tumors. *Cancer Metastasis Rev* **3**: 115–126

Tokuda K, Abe H, Aida T, Sugimoto S and Kaneko S (1990) Modification of tumor blood flow and enhancement of therapeutic effect of ACNU on experimental rat gliomas with angiotensin II. *J Neurooncol* **8**: 205–212

Tozer GM and Shaffi KM (1993) Modification of tumour blood flow using the hypertensive agent angiotensin II. *Br J Cancer* **67**: 981–988

Tozer GM and Shaffi KM (1995) The response of tumour vasculature to angiotensin II revealed by its systemic and local administration to “tissue-isolated” tumours. *Br J Cancer* **72**: 595–600

Tozer GM, Shaffi KM, Prise VE and Bell KM (1996) Spatial heterogeneity of tumour blood flow modification induced by angiotensin II: Relationship to receptor distribution. *Int J Cancer* **65**: 658–663

Trotter MJ, Chaplin DJ and Olive PL (1991) Effect of angiotensin II on intermittent tumour blood flow and acute hypoxia in the murine SCCVII carcinoma. *Eur J Cancer* **27**: 887–893

UKCCCR (1998) United Kingdom Co-ordinating Committee on Cancer Research guidelines for the welfare of animals in experimental neoplasia (2nd edn). *Br J Cancer* **77**: 1–10

Vaupel PW (1993) Oxygenation of solid tumors. In *Drug Resistance in Oncology* Teicher BA (ed) pp 53–85. Marcel Dekker: New York

Vaupel P, Kelleher DK, Thews O and Hummel M (2000) Tumour blood flow: critical issues and modification. In: Moriarty M, Mothersill C, Seymour C, Edington M, Ward JF, Fry RJM (eds) Radiation Research, Vol. 2 (Proceedings, 11th International Congress on Radiation Research). Allen Press, Lawrence

Vaupel P and Gabbert H (1986) Evidence for and against a tumor type-specific vascularity. *Strahlenther Onkol* **162**: 633–638

Vaupel P and Mueller-Klieser W (1986) Cell line and growth site as relevant parameters governing tumor tissue oxygenation. *Adv Exp Med Biol* **200**: 633–643

Vaupel P, Kallinowski F and Okunieff P (1989) Blood flow, oxygen and nutrient supply, and metabolic microenvironment of human tumors: a review. *Cancer Res* **49**: 6449–6465

Vaupel P, Kelleher DK and Thews O (1998) Modulation of tumor oxygenation. *Int J Radiat Oncol Biol Phys* **42**: 843–848

Vaupel P, Schlenger K, Knoop C and Höckel M (1991) Oxygenation of human tumors: Evaluation of tissue oxygen distribution in breast cancers by computerized $O_2$ tension measurements. *Cancer Res* **51**: 3316–3322

Warren BA (1979) The vascular morphology of tumors. In *Tumor Blood Circulation*, Peterson HI (ed) pp 1–47 CRC Press: Boca Raton

© 2000 Cancer Research Campaign
